# A Narrative Review on Plasminogen Activator Inhibitor-1 and Its (Patho)Physiological Role: To Target or Not to Target?

**DOI:** 10.3390/ijms22052721

**Published:** 2021-03-08

**Authors:** Machteld Sillen, Paul J. Declerck

**Affiliations:** Laboratory for Therapeutic and Diagnostic Antibodies, Department of Pharmaceutical and Pharmacological Sciences, KU Leuven, B-3000 Leuven, Belgium; machteld.sillen@kuleuven.be

**Keywords:** plasminogen activator inhibitor-1, PAI-1, fibrinolysis, cardiovascular disease, cancer, inflammation, fibrosis, aging

## Abstract

Plasminogen activator inhibitor-1 (PAI-1) is the main physiological inhibitor of plasminogen activators (PAs) and is therefore an important inhibitor of the plasminogen/plasmin system. Being the fast-acting inhibitor of tissue-type PA (tPA), PAI-1 primarily attenuates fibrinolysis. Through inhibition of urokinase-type PA (uPA) and interaction with biological ligands such as vitronectin and cell-surface receptors, the function of PAI-1 extends to pericellular proteolysis, tissue remodeling and other processes including cell migration. This review aims at providing a general overview of the properties of PAI-1 and the role it plays in many biological processes and touches upon the possible use of PAI-1 inhibitors as therapeutics.

## 1. Introduction

**Figure 1:**
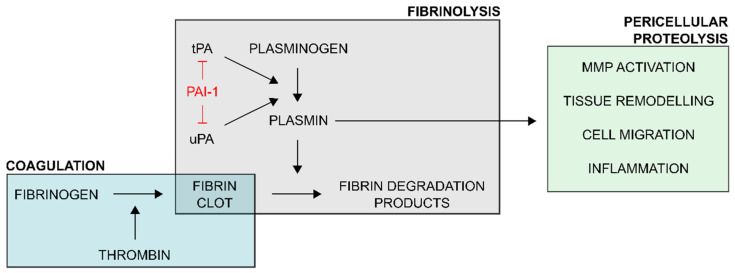
Schematic overview of the regulatory role of plasminogen activator inhibitor-1 (PAI-1) in the plasminogen activator/plasmin system. Upon vascular injury, the coagulation process ultimately generates thrombin which acts upon fibrinogen to form an insoluble fibrin clot. This coagulatory response is balanced by the fibrinolytic system. Plasminogen activators (PAs) tissue-type PA (tPA) and urokinase-type PA (uPA) convert plasminogen to proteolytically active plasmin. Plasmin, the key enzyme of the fibrinolytic system, degrades the fibrin clot into soluble fibrin degradation products. Through uPA-mediated plasminogen activation, the function of plasmin extends to pericellular proteolytic processes, involving activation of matrix metalloproteinases (MMPs), tissue remodeling, cell migration and adhesion, and inflammation. PAI-1 is an important regulator of the plasminogen activator/plasmin system as it interferes with plasminogen activation by directly inhibiting tPA and uPA.

Plasminogen activator inhibitor-1 (PAI-1) belongs to the family of serine protease inhibitors (serpins) and is an important regulator of the plasminogen/plasmin system (Figure 1) [1]. This system revolves around the conversion of the zymogen plasminogen into the active enzyme plasmin through proteolytic cleavage that is mediated by plasminogen activators (PAs). When mediated by tissue-type PA (tPA), plasmin is primarily involved in fibrinolysis as it degrades the insoluble fibrin meshwork that constitutes blood clots. Through urokinase-type PA (uPA)-mediated plasminogen activation, the function of the plasminogen/plasmin system extends to pericellular proteolysis associated with processes including tissue remodeling and cell migration. Since its discovery, the (patho)physiological role of PAI-1 has been extensively studied in humans as well as in diverse disease models in animals. A link has been demonstrated between PAI-1 and various diseases including cardiovascular disease (CVD), metabolic disturbances, aging, cancer, tissue fibrosis, inflammation, and neurodegenerative disease. As a consequence, several PAI-1 inhibitors have been developed to further study the role of PAI-1 in disease models and to explore their potential applications in a therapeutic setting. This narrative review aims at providing a general overview of the properties of PAI-1 and the role it plays in many biological processes and touches upon the possible use of PAI-1 inhibitors as therapeutics.

## 2. PAI-1 Synthesis and Distribution

PAI-1 is the primary physiological inhibitor of plasminogen activators tPA and uPA. It was first detected almost four decades ago as an inhibitor of the fibrinolytic system produced by cultured bovine endothelial cells [2], but can be expressed by several other cell types in various tissues [3,4,5]. The expression and release of PAI-1 is strongly regulated by various factors, including growth factors, inflammatory cytokines, hormones, glucose, and endotoxins [6,7]. PAI-1 is synthesized as a 45-kDa single-chain glycoprotein comprising 379 or 381 amino acids depending on alternative cleavage sites for signal peptidases [8]. Three potential glycosylation sites have been identified based on the amino-acid sequence, of which Asn209 and Asn265 display a tissue-type specific glycosylation pattern [9]. In the blood, PAI-1 circulates in two distinct pools, free in plasma or retained in platelets [10]. Even though plasma PAI-1 circulates at relatively low levels (5–50 ng/mL), it mainly adopts the active conformation. In contrast, the main blood pool of PAI-1 is retained in platelets (up to approximately 300 ng/mL) and was found to be only 2–5% functionally active upon lysis of platelets [11,12]. However, more recent studies demonstrated that upon platelet activation platelet-derived PAI-1 may be present in the active conformation at significantly higher levels [13,14], presumably due to de novo PA-1 synthesis through translationally active PAI-1 messenger RNA of which the synthesis rate is importantly upregulated by platelet activation [13]. Furthermore, platelet activation results in the release of PAI-1 followed by partial retention of PAI-1 on the platelet membrane, thereby contributing to thrombolysis resistance of the clot [14,15,16].

## 3. PAI-1 Structure and Function

### 3.1. PAI-1 Is an Inhibitory Serpin

As a member of the serpin superfamily, PAI-1 displays their common highly ordered structure that is characterized by three β-sheets (A, B, and C) and nine α-helices (hA through hI) [17,18]. PAI-1 is synthesized in a metastable active conformation, exposing a flexible reactive center loop (RCL) at the top of the molecule. The RCL comprises 26 residues (designated P16 to P10′), including a bait peptide bond that mimics the normal substrate of the PAs (designated P1-P1′).

Upon interaction, the PA binds to PAI-1 through the P1-P1′ reactive center and several exosite regions adjacent to the RCL to form a noncovalent Michaelis complex. Subsequently, the PA active site serine (tPA-Ser478 or uPA-Ser195) attacks the P1-P1′ bond to form a tetrahedral intermediate with PAI-1. Successful cleavage of this bond yields the acyl-enzyme intermediate in which the PA is covalently linked to the P1 residue in PAI-1. The PAI-1/PA reaction then follows a branched pathway mechanism in which PAI-1 either acts as an inhibitor or a substrate towards the PA.

In the inhibitory pathway, the acyl-enzyme intermediate is converted to an irreversible inhibitory complex by full insertion of the N-terminal part of the RCL (P16-P1) as strand 4 into the central PAI-1 β-sheet A. Simultaneously, the PA is translocated to the opposite side of the PAI-1 molecule where a large part of the PA is deformed by compression against the body of PAI-1 [19,20]. Deformation of the PA and especially its active site prevents hydrolysis of the acyl-enzyme intermediate and traps the PA as a stable PAI-1/PA complex.

In contrast, the substrate pathway is characterized by hydrolysis of the acyl-enzyme intermediate prior to PA distortion, resulting in the release of regenerated PA from cleaved PAI-1 [21,22]. Substrate behavior of PAI-1 has been associated with either the existence of a conformational distinct substrate-like subset of PAI-1 [23,24], or can be induced by changing the kinetic parameters that underlie the branched pathway mechanism in favor of the substrate pathway [25,26].

### 3.2. PAI-1 Stability

PAI-1 is unique among serpins as active PAI-1 spontaneously converts into a thermodynamically stable latent form by slowly self-inserting the N-terminal part of the RCL into the core of the PAI-1 molecule. As a result, the P1-P1′ reactive center becomes inaccessible for PAs [27]. Whereas this transition occurs with a functional half-life of approximately two hours at 37 °C in vitro, the active form of PAI-1 is stabilized at least two-fold in vivo by the association with vitronectin in plasma and the extracellular matrix [28,29]. Furthermore, external conditions (ionic strength [29,30], pH [29], metal ions [31], arginine [32,33]), binding to other proteins (α_1_-acid glycoprotein [34], antibodies [35]), and mutagenesis [36] have been shown to also affect the stability of PAI-1 (reviewed elsewhere [37]).

### 3.3. Interactions with Non-Proteinase Ligands

Apart from binding and inactivating PAs, PAI-1 can also interact with non-proteinase ligands including vitronectin and members of the low-density lipoprotein receptor (LDLR) family. Through these interactions, the functions of PAI-1 extend to various (patho)physiological processes not relying on its anti-protease activity.

As mentioned, active PAI-1 is importantly stabilized through allosteric modulation by its high-affinity interaction with the aminoterminal somatomedin B (SMB) domain of the glycoprotein vitronectin. Vitronectin is abundantly present in plasma (~300 µg/mL) and the extracellular matrix and plays a pivotal role in tissue remodeling, cell differentiation and migration, and inflammation. These effects are mediated by binding of the SMB domain or the neighboring Arg-Gly-Asp (RGD) motif [38,39] of vitronectin to cell surface-associated proteins including integrins and transmembrane receptors such as the uPA receptor (uPAR), which initiates intracellular signaling events. Due to the proximity of the binding sites for PAI-1, integrins, and uPAR, PAI-1 can interfere with vitronectin function as it competes with integrins and uPAR for binding to vitronectin.

Adjacent to the vitronectin binding site, PAI-1 also displays a high-affinity binding site for receptors of the LDLR family, such as LDLR-related protein 1 (LRP1) and the very-low-density lipoprotein receptor [40]. Whereas cleaved, latent, and native PAI-1 depend on LRP1 for cellular clearance, clearance of the PAI-1/uPA complex is greatly enhanced by the involvement of uPAR. By interacting with uPA, which is in turn associated with the cell surface uPAR, PAI-1 is localized to the cell-surface, thereby facilitating the interaction between the PAI-1 moiety within the PAI-1/uPA/uPAR complex and LRP1. Subsequently, internalization of the complex through endocytosis results in the degradation of the PAI-1/uPA complex and recycling of free uPAR to the cell surface. Since both the uPAR and LDLR receptors are important for intracellular signaling as well, binding of PAI-1 or the PAI-1/uPA complex can either indirectly affect signaling activity by regulating receptor levels on the cell surface, or directly by inducing receptor activity through a direct binding interaction.

## 4. Role of PAI-1 in Diverse Pathologies

The role of PAI-1 in diverse (patho)physiological processes has been extensively studied in humans, in mice being genetically or functionally deficient for PAI-1, or in transgenic mice overexpressing PAI-1. A link has been demonstrated between PAI-1 and various diseases including cardiovascular disease (CVD), metabolic disturbances, aging, cancer, tissue fibrosis, inflammation, and neurodegenerative disease.

### 4.1. PAI-1 in Cardiovascular Disease

Hemostasis is an important physiological process for maintaining vascular integrity and securing a sufficient blood flow throughout the circulatory system. Therefore, it requires a dynamic interplay between the vascular system, blood platelets, the coagulatory system, and the fibrinolytic system. As PAI-1 is a major inhibitor of the fibrinolytic system, elevated PAI-1 levels create a hypofibrinolytic or prothrombotic state that may contribute to the development of CVD. The recent 2018 report issued by the World Health Organization [41] once more underscores the high mortality rate for cardiovascular diseases, accounting for an estimated 17.8 million deaths worldwide in 2016. An important fraction, i.e., 87% of the deaths caused by CVD, can be contributed to ischemic heart disease (IHD) and stroke. Both IHD and stroke occur when the blood supply to either the heart or the brain is insufficient due to blockage of the blood vessels supplying the organ. This blockage is often caused by the formation of a blood clot (thrombosis) or a buildup of plaque (atherosclerosis), two processes that often coincide with increased plasma PAI-1 levels. Transgenic mice overexpressing wild-type or a stabilized active mutant of human PAI-1 were shown to develop either transient venous thrombosis or age-dependent coronary arterial thrombosis and myocardial infarction, respectively [42,43]. Myocardial infarction is often caused by occlusive thrombus formation that is triggered by the exposure of a procoagulatory surface following disruption of an atherosclerotic plaque in the coronary arteries [44]. Several cell types associated with atherosclerotic plaques in human coronary arteries have been shown to overexpress PAI-1, with the highest levels found in the vulnerable part of the plaque [45,46,47,48]. Through local inhibition of plasmin generation, PAI-1 can reduce tissue remodeling and potentially stabilize the fibrin matrix of the developing plaque. Furthermore, studies on the effects of pharmacological PAI-1 inhibition on atherogenesis in mice with obesity and metabolic syndrome also suggested a role for PAI-1 in adipose tissue inflammation, macrophage accumulation, and inducing senescence of smooth muscle cells through its interaction with LRP1 [49]. Whereas several studies demonstrated substantial evidence for PAI-1 as an independent risk factor for CVD including myocardial infarction and stroke [50,51,52], coronary heart disease [53], venous thrombosis [54], and atherosclerosis [45,55], other studies could not confirm these associations or the significance for the link was lost after adjusting for other risk factors, such as age, sex, and metabolic abnormities [52,56,57,58].

### 4.2. PAI-1 in Metabolic Disturbances

Several epidemiological studies have demonstrated that elevated circulating PAI-1 levels and PAI-1 activity are an important feature of or even a marker for the development of metabolic disturbances including obesity, type 2 diabetes, and metabolic syndrome [59,60,61]. Metabolic syndrome is a multifactorial disease characterized by a cluster of co-occurring metabolic abnormalities that include central obesity, impaired glucose tolerance, hyperinsulinemia, dyslipidemia, and hypertension. Furthermore, these symptoms are all well-documented risk factors for cardiovascular disease and diabetes [62] and it has been shown that individuals with the metabolic syndrome are at higher risk for developing these comorbidities [63]. Importantly, elevated levels of glucose [64] and insulin [65,66] and its precursors [67], and free fatty acids [66,68] have been shown to induce PAI-1 expression or to reduce the rate of PAI-1 mRNA degradation [69]. Moreover, it was demonstrated in obese mice [70] and later confirmed in human adipose tissue [71] that adipocytes are an important source of PAI-1, underscoring the contribution of enlarged adipose tissue to circulating PAI-1 levels. On the other hand, obesity, type 2 diabetes, and metabolic syndrome are often associated with a chronic state of inflammation that is characterized by overexpression of inflammatory adipokines, such as interleukin-6 (IL-6) and tumor necrosis factor-α (TNF-α) [72], which induce PAI-1 expression in adipose tissue [73,74]. These increased PAI-1 levels further contribute to the development of inflammation in adipose tissue by increasing the number of inflammatory macrophages that infiltrate in the tissue [75]. Apart from the positive correlation between inflammatory markers and PAI-1 levels, a link has also been observed between PAI-1 and lipid metabolism in obesity [76,77]. In this respect, higher PAI-1 levels coincide with a decreased mean low-density lipoprotein (LDL) size and higher amounts of small-dense LDL lipoprotein fraction, which importantly contribute to the atherogenic lipid profile and increased cardiovascular risk in obesity [76]. Of note, several studies have shown that PAI-1 deficiency [78,79,80], pharmacological inhibition of PAI-1 [81], and a reduction in plasma PAI-1 levels through dietary restrictions [82] are protective against the development of obesity and metabolic disorders.

### 4.3. PAI-1 in Inflammation and Infectious Disease

Acute phase proteins, such as PAI-1, play an important role in inflammatory and immune responses following infectious and/or noninfectious injuries. Several studies have shown that PAI-1 is a critical mediator of the early host defense response that is necessary for the eradication of various pathogens [83,84,85]. In contrast, uncontrolled inflammation is an essential component of several diseases including respiratory diseases, such as acute respiratory distress syndrome (ARDS) caused by bacterial or viral infections or sepsis [86]. Two characteristic features of ARDS, namely the formation of intravascular micro-thrombi and fibrin deposits in the alveolar space, are often the combined result of tissue factor generated by inflammatory cells (procoagulatory) and PAI-1 produced by endothelial cells (antifibrinolytic) [87]. Recently, it was shown that, in patients with severe coronavirus disease 2019 (COVID-19), plasma PAI-1 levels were as highly elevated as compared to patients with bacterial sepsis or ARDS [88]. As mentioned, expression of PAI-1 can be induced by a wide range of pro-inflammatory mediators including IL-6 and TNF-α, which are known as components of the cytokine release syndrome that is observed in many patients with severe COVID-19 [89]. Another study revealed that elevated levels of the PAI-1/tPA complex were related with disease severity and could be considered an independent risk factor for death in patients with COVID-19 [90]. By affecting multiple organs, including the lung as the predominant target, the formation of microthrombi in these organs may lead to multiorgan failure [91]. In this respect, the efficacy, i.e., an improved clinical outcome, a lower degree of organ failure, decreased PAI-1 levels, and ventilator free days, as well as the safety of a small molecule PAI-1 inhibitor, TM5614, are currently investigated in a phase 1/2 clinical trial for high-risk patients hospitalized with severe COVID-19 (ClinicalTrials.gov: NCT04634799).

### 4.4. PAI-1 in Cancer

Since motile cells focalize uPA on the cell surface through association with uPAR, which is highly expressed on tumor cells, uPA has been considered as the critical trigger for plasmin formation during tumor cell invasion and metastasis. Importantly, by having binding sites other than for uPA, uPAR interacts with vitronectin, integrins, and transmembrane receptors to facilitate intracellular signaling by effector molecules that are involved in cell migration [92]. By binding to vitronectin, PAI-1 prevents the interaction between vitronectin and two cell surface-associated proteins, namely uPAR and α_v_β_3_ integrin, and as a result represses cell migration on vitronectin in the extracellular matrix (ECM). Apart from directly inhibiting uPA-mediated plasmin formation, PAI-1 also inhibits the activity of the uPA/uPAR complex by promoting its endocytosis via LRP1, followed by the degradation of uPA and recycling of uPAR. Furthermore, it causes the cell to detach from the ECM. PAI-1 was thus expected to have an anti-tumor effect. Interestingly, ample evidence has been provided for a paradoxical pro-tumorigenic function of PAI-1, being both pro-angiogenic [93] and anti-apoptotic [94], documented to be dependent on the stage of cancer progression, the cell type, the source (i.e., host or tumor) and on the relative concentration of PAI-1 [93,95,96,97,98]. By blocking α_v_β_3_-mediated endothelial cell migration on vitronectin in the extracellular matrix, PAI-1 was shown to promote angiogenesis by stimulating integrin α_5_β_1_-mediated endothelial cell migration toward fibronectin inside tumor tissue [99]. Apart from its interaction with vitronectin, PAI-1 can modulate cell migration by binding to surface receptor LRP1 that triggers intracellular signaling.

Indeed, uPA and PAI-1 are among the most highly induced proteins in several migratory or invasive tumor cell types. Even though some studies failed to show a correlation between elevated levels of PAI-1 and poor clinical prognosis [100,101,102], PAI-1 has been established as one of the most reliable biomarkers and prognostic markers in many cancer types, including breast [103,104,105,106], ovarian [107], bladder [108,109], colon [110], renal [111] and non-small cell lung cancers [112].

### 4.5. PAI-1 in Fibrosis

The plasminogen activator/plasmin system is also important for tissue remodeling and promoting wound healing, which is characterized by inflammation, cellular migration to the wound area, and the activation and differentiation of fibroblasts, and the synthesis of ECM proteins to heal the wound [113]. As PAI-1 inhibits PA-mediated plasmin formation and thus protects ECM proteins from degradation, it facilitates wound healing. However, excessive and sustained PAI-1 activity promotes excess fibrin accumulation and leads to low ECM degradation which results in excessive collagen accumulation. Several studies showed that PAI-1 contributes to tissue fibrosis which can affect multiple organs including the skin [114,115], lungs [116,117], kidneys [118], and liver [119]. In agreement with these reports, pharmacological inhibition of PAI-1 or deficiency of host PAI-1 accelerate wound healing [120] and attenuate fibrosis [121,122,123,124,125]. In contrast, complete PAI-1 deficiency in mice leads to spontaneous development of cardiac fibrosis in older animals. The observed accelerated fibrosis has been shown to be the result of an increased vascular permeability, local inflammation, and excessive ECM remodeling, caused by the lack of PAI-1-mediated regulation of integrin α_v_β_3_ and a consequently increased signaling of transforming growth factor-β, a potent profibrotic molecule [126,127].

### 4.6. PAI-1 in the Central Nervous System

PAI-1 is also produced in brain tissue where it has an anti-apoptotic role in neurons by acting as an inhibitor to tPA. Alternatively, PAI-1 protects neurons by preventing disintegration of neuronal networks by maintaining or promoting neuroprotective signaling, independent from its function as a proteinase inhibitor [128]. PAI-1 has also been shown to promote the migration of microglial cells, which are the resident macrophages in the central nervous system, through an LRP1-dependent mechanism, as well as to modulate their phagocytic activity via a vitronectin-dependent mechanism [129]. However, when chronically activated, microglia also contribute to neurodegenerative diseases by maintaining neuroinflammation. Several reports have indicated a role of PAI-1 in central nervous system pathology, including multiple sclerosis [130,131], Alzheimer’s disease [132,133], and Parkinson’s disease [134,135]. In demyelinated axons in inflammatory multiple sclerosis lesions, increased PAI-1 levels impair the capacity of the tPA/plasmin system to clear fibrin(ogen) deposits and therefore contribute to axonal damage in multiple sclerosis [131,136]. Likewise, elevated levels of PAI-1 have been shown to interfere with the plasmin-mediated clearance and degradation of amyloid-β (Aβ), thereby contributing to the deposition of Aβ into neurotoxic amyloid plaques and dementia in Alzheimer’s disease. Furthermore, inhibition of PAI-1 in transgenic Aβ-producing mice significantly lowered plasma and brain Aβ levels and reversed the cognitive deficits [133,137]. Recently, it was hypothesized that the synergistic relationship between α-synuclein, aggregation and neuroinflammation up-regulate the expression of PAI-1, suggesting a pathological amplification loop, i.e., increased α-synuclein aggregation results in an inflammatory response from microglia, a subsequent increase in PAI-1 levels and thus a decrease in plasmin formation, leading to the accumulation of α-synuclein and a further amplified inflammatory response [135].

### 4.7. PAI-1 in Aging

Age is the largest risk factor for most chronic diseases, including CVD, metabolic syndrome, and type 2 diabetes. On a cellular level, senescence is a process characterized by the loss of normal physiological function and permanent growth arrest, which accelerates organ and systemic aging when induced by, e.g., oxidative stress. Several molecular drivers of aging have been proposed, including shortening of the telomere length, genomic instability, loss of proteostasis, and altered intercellular communication [138]. Senescent cells have been shown to secrete bioactive molecules called the senescence-associated secretory phenotype (SASP) [139]. These factors have been shown to modulate not only the functions of the secreting cells but also those of adjacent cells. Importantly, PAI-1 levels have been reported to increase with age in various tissues and PAI-1 has been identified as a fundamental component of the SASP, being part of the signaling circuit that induces senescence in neighboring cells [140]. Recently, in the Berne Amish kindred, carriers of the null *SERPINE1* allele, i.e., a rare loss-of-function mutation in the *SERPINE1* gene that encodes PAI-1 which is associated with a lifelong reduction in PAI-1 levels, were shown to have a longer life span [141]. The same study identified an association between heterozygosity of the null *SERPINE1* and a longer leukocyte telomere length, a better metabolic profile and a lower prevalence of diabetes. Therefore, PAI-1 may act not only as a marker but also as a mediator of cellular senescence associated with aging and aging-related pathologies [142].

## 5. Diverse Approaches to Inhibit PAI-1

From the various biological roles of PAI-1 and its contribution to a wide variety of pathological processes it is clear that targeting PAI-1 may have significant beneficial effects. Therefore, many efforts have been devoted to the development of selective PAI-1 inhibitors, in particular for the prevention or treatment of cardiovascular disease. Some marketed drugs, including insulin-sensitizing agents [143] and angiotensin-converting enzyme inhibitors [144], and antisense oligonucleotides have been shown to attenuate PAI-1 synthesis or secretion [145]. In contrast, the majority of PAI-1 inhibitors currently in development (extensively reviewed elsewhere [37,146,147] can influence PAI-1 functionality in at least four possible ways, i.e., (I) by blocking the interaction between PAI-1 and PAs, (II) by inducing substrate behavior of PAI-1, (III) by accelerating the active-to-latent transition or converting active PAI-1 to an otherwise inert form, or (IV) by interfering with interactions between PAI-1 and other biological ligands such as LRP1. These inhibitors include small molecules, peptides, antibodies (Abs), and antibody fragments such as nanobodies. A link between the mechanisms by which these inhibitors modulate PAI-1 functionality and their binding site has been provided by using a broad range of biochemical and biophysical methods, including mutagenesis studies, competitive binding experiments, computational docking, and X-ray crystallography.

PAI-1 inhibitory peptides have been shown to either induce substrate behavior of PAI-1 or to accelerate the conversion to an inert form of PAI-1. Synthetic peptides that were derived from the sequence of the RCL were shown to insert into the core of the PAI-1 protein in between strand 3 and strand 5 of the central β-sheet A. It was suggested that, depending on their position within the cleft, i.e., occupying the same space as the N-terminal part or the C-terminal part of the RCL in latent or cleaved PAI-1, they act by inducing substrate behavior of PAI-1 or by accelerating the irreversible transition to inert PAI-1, respectively [148]. In contrast, a peptide that was isolated from a phage-display peptide library, paionin-4, was shown to accelerate the active-to-latent conversion by binding to a different region in PAI-1, located at the loop between hD and s2A [149]. From the same library, the peptide paionin-1 did not affect PAI-1 activity; however, it was able to prevent the binding of the PAI-1/uPA complex to LRP1 by binding hD and hE in the flexible joint region of PAI-1, which may impair the signaling function of uPA/uPAR/LRP1 [150].

**Figure 2:**
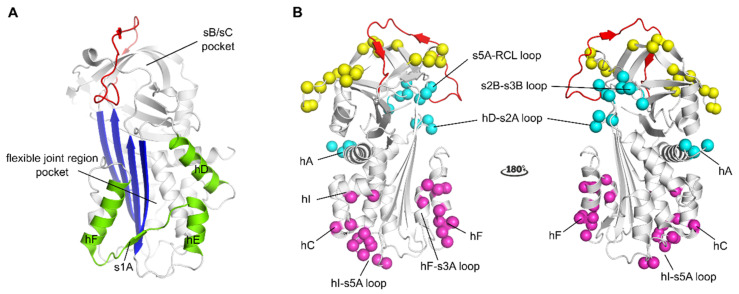
Localization of binding regions for PAI-1 inhibitors in the structure of active PAI-1. (**A**) Localization of the binding regions for small molecule PAI-1 inhibitors. The binding pocket in the flexible joint region is aligned by hD, hE, hF and strand 1 (shown in green). The sB/sC pocket is aligned by β-sheet B and C (**B**), localization of different epitopes of antibodies and antibody fragments as determined by mutagenesis and X-ray crystallographic studies. The epitopes of Abs that prevent the interaction between PAI-1 and PAs comprise residues that are indicated by yellow spheres (exosites for PAs on PAI-1) and residues in the reactive center loop (RCL) (shown in red). The epitopes of switching Abs are indicated by magenta spheres and comprise either residues located in hF and the loop connecting hF and s3A or residues located in the loop connecting hI and s5A, hC and hI. The epitopes of latency-inducing Abs are indicated by cyan spheres and comprise in hA or residues at the top part of the PAI-1 molecule in the hD-s2A loop, the s2B-s3B loop, and the s5A-RCL loop. All panels have been generated using the structure of active PAI-1 (PDB ID 1DB2).

Another large category of PAI-1 inhibitors includes small organochemical molecules that are very diverse in their chemical structure. Many of these compounds have been shown to bind a common binding pocket within the area of the flexible joint region of PAI-1 [151,152,153], or to link structural elements within this region through interactions at the PAI-1 surface [154] (Figure 2A). By interfering with the flexible joint region, these compounds were shown to inhibit PAI-1 through a dual mechanism of action, i.e., by inducing substrate behavior of PAI-1 and converting PAI-1 to an inert form which can be latent or unreactive PAI-1 or PAI-1 in the capacity of polymers. By binding this otherwise flexible region in PAI-1, these compounds can induce substrate behavior possibly by attenuating or preventing the conformational rearrangements within this region that are required for a successful inhibitory reaction between PAI-1 and PA’s or by affecting regions outside the flexible joint region through allosteric modulation. In contrast to the aforementioned compounds, compounds that bind the sheet B/sheet C (sB/sC) pocket (Figure 2A), i.e., an interface composed of residues from the s3A/s4C loop, β-sheets B and C, and hH, were shown to block initial PAI-1/PA Michaelis complex formation, possibly by a reversible allosteric modulation of the RCL [155].

In contrast to inhibitory peptides and small molecules, the binding sites of antibody-based PAI-1 inhibitors have been mapped to different regions of the PAI-1 molecule (extensively reviewed in [37]). Overall, Abs that interfere with PAI-1 activity can be divided into three categories. The first category of Abs or antibody fragments acts by interfering with the formation of the initial Michaelis complex. It was recently shown that PAI-1/PA complex formation can be prevented by destabilizing the Michaelis complex merely by hampering exosite interactions between PAI-1 and PAs or in combination with shielding the P1-P1′ reactive center in the RCL of PAI-1 (Figure 2B). The second category comprises Abs and antibody fragments that switch the PAI-1/PA reaction towards the substrate pathway. These Abs, referred to as “switching antibodies”, can bind different epitope regions in the lower half of the PAI-1 molecule. Within this category, Abs that bind hF or the loop connecting hF to s3A of the central PAI-1 β-sheet A (Figure 2B) were shown to slow down the rate of cleaved RCL insertion, resulting in hydrolysis of the PAI-1/PA complex. On the other hand, Abs that bind the loop between hI and s5A at the bottom of the PAI-1 molecule, a region that is buried by the PA in the final inhibitory PAI-1/PA complex, hinder full translocation of the PA and thus prevent distortion of the catalytic triad of the PA. The third category of Abs have the ability to accelerate the active-to-latent transition of PAI-1 and were shown to bind different epitopes that are spread more across the PAI-1 surface (Figure 2B). In most cases, these epitopes comprise regions at the top of the PAI-1 molecule that are less accessible in the active form of PAI-1. Binding is therefore believed to occur to a prelatent state of PAI-1, in which the RCL is already partially inserted. On the other hand, acceleration of the active-to-latent transition was also observed for an Ab binding to the N-terminal part of hA in rat PAI-1. However, an Ab targeting as similar region in human PAI-1 was shown to be non-inhibitory.

Even though several of these molecules were proven to be efficient PAI-1 inhibitors both in vitro and in vivo, no PAI-1 inhibitor is currently approved for therapeutic use in humans. However, it should be noted that a few PAI-1 antagonists are currently proceeding through clinical trials, which underscores the clinical interest in safe and efficient modulators of PAI-1 activity for the treatment of PAI-1-related diseases.

## Abbreviations

Aβ	Amyloid-β
Abs	Antibodies
ARDS	Acute respiratory distress syndrome
COVID-19	Coronavirus disease 2019
CVD	Cardiovascular disease
ECM	Extracellular matrix
IHD	Ischemic heart disease
IL-6	Interleukin-6
LDL	Low-density lipoprotein
LDLR	Low-density lipoprotein receptor
LRP1	LDLR-related protein 1
MMP	Matrix metalloprotease
PA	Plasminogen activator
PAI-1	Plasminogen activator inhibitor-1
RCL	Reactive center loop
SASP	Senescence-associated secretory phenotype
SMB	Somatomedin B
TNF-α	Tumor necrosis factor-α [72]
tPA	Tissue-type plasminogen activator
uPA	Urokinase-type plasminogen activator
uPAR	uPA receptor

## References

[1] Gils A., Declerck P.J. (2013). Three Decades of Research on Plasminogen Activator Inhibitor-1: A Multifaceted Serpin. Semin. Thromb. Hemost..

[2] Loskutoff D.J., Van Mourik J.A., Erickson L.A., Lawrence D. (1983). Detection of an unusually stable fibrinolytic inhibitor produced by bovine endothelial cells. Proc. Natl. Acad. Sci. USA.

[3] Chmielewska J., Rånby M., Wiman B. (1983). Evidence for a rapid inhibitor to tissue plasminogen activator in plasma. Thromb. Res..

[4] Simpson A.J., Booth N.A., Moore N.R., Bennett B. (1991). Distribution of plasminogen activator inhibitor (PAI-1) in tissues. J. Clin. Pathol..

[5] Crandall D.L., Quinet E.M., Morgan G.A., Busler D.E., McHendry-Rinde B., Kral J.G. (1999). Synthesis and Secretion of Plasminogen Activator Inhibitor-1 by Human Preadipocytes. J. Clin. Endocrinol. Metab..

[6] Rabieian R., Boshtam M., Zareei M., Kouhpayeh S., Masoudifar A., Mirzaei H. (2018). Plasminogen Activator Inhibitor Type-1 as a Regulator of Fibrosis. J. Cell. Biochem..

[7] Yamamoto K., Takeshita K., Shimokawa T., Yi H., Isobe K.-I., Loskutoff D.J., Saito H. (2002). Plasminogen activator inhibitor-1 is a major stress-regulated gene: Implications for stress-induced thrombosis in aged individuals. Proc. Natl. Acad. Sci. USA.

[8] Andreasen P., Riccio A., Welinder K., Douglas R., Sartorio R., Nielsen L., Oppenheimer C., Blasi F., Danø K. (1986). Plasminogen activator inhibitor type-1: Reactive center and amino-terminal heterogeneity determined by protein and cDNA sequencing. FEBS Lett..

[9] Gils A., Pedersen K.E., Skottrup P., Christensen A., Naessens D., Deinum J., Enghild J.J., Declerck P.J., Andreasen P.A. (2003). Biochemical importance of glycosylation of plasminogen activator inhibitor-1. Thromb. Haemost..

[10] Booth N.A., Simpson A.J., Croll A., Bennett B., MacGregor I.R. (1988). Plasminogen activator inhibitor (PAI-1) in plasma and platelets. Br. J. Haematol..

[11] Booth N., Cröll A., Bennett B. (1990). The activity of plasminogen activator inhibitor-1 (PAI-1) of human platelet. Fibrinolysis.

[12] Declerck P.J., Alessi M.C., Verstreken M., Kruithof E.K., Juhan-Vague I., Collen D. (1988). Measurement of plasminogen activator inhibitor 1 in biologic fluids with a murine monoclonal antibody-based enzyme-linked immunosorbent assay. Blood.

[13] Brogren H., Wallmark K., Deinum J., Karlsson L., Jern S. (2011). Platelets Retain High Levels of Active Plasminogen Activator Inhibitor 1. PLoS ONE.

[14] Morrow G.B., Whyte C.S., Mutch N.J. (2019). Functional plasminogen activator inhibitor 1 is retained on the activated platelet membrane following platelet activation. Haematology.

[15] Torr-Brown S.R., Sobel B.E. (1993). Attenuation of thrombolysis by release of plasminogen activator inhibitor type-1 from platelets. Thromb. Res..

[16] Stringer H.A., Van Swieten P., Heijnen H.F., Sixma J.J., Pannekoek H. (1994). Plasminogen activator inhibitor-1 released from activated platelets plays a key role in thrombolysis resistance. Studies with thrombi generated in the Chandler loop. Arterioscler. Thromb. J. Vasc. Biol..

[17] Irving J.A., Pike R.N., Lesk A.M., Whisstock J.C. (2000). Phylogeny of the Serpin Superfamily: Implications of Patterns of Amino Acid Conservation for Structure and Function. Genome Res..

[18] Gettins P.G., Olson S.T. (2016). Inhibitory serpins. New insights into their folding, polymerization, regulation and clearance. Biochem. J..

[19] Huntington J.A., Read R.J., Carrell R.W. (2000). Structure of a serpin–protease complex shows inhibition by deformation. Nat. Cell Biol..

[20] Perron M.J., Blouse G.E., Shore J.D. (2003). Distortion of the Catalytic Domain of Tissue-type Plasminogen Activator by Plasminogen Activator Inhibitor-1 Coincides with the Formation of Stable Serpin-Proteinase Complexes. J. Biol. Chem..

[21] Aertgeerts K., De Bondt H.L., De Ranter C.J., Declerck P.J. (1995). Mechanisms contributing to the conformational and functional flexibility of plasminogen activator inhibitor-1. Nat. Genet..

[22] Dewilde M., Strelkov S., Rabijns A., Declerck P. (2009). High quality structure of cleaved PAI-1-stab. J. Struct. Biol..

[23] Declerck P., De Mol M., Vaughan D., Collen D. (1992). Identification of a conformationally distinct form of plasminogen activator inhibitor-1, acting as a noninhibitory substrate for tissue-type plasminogen activator. J. Biol. Chem..

[24] Urano T., Strandberg L., Johansson L.B.-A., Ny T. (1992). A substrate-like form of plasminogen-activator-inhibitor type 1. Conversions between different forms by sodium dodecyl sulphate. JBIC J. Biol. Inorg. Chem..

[25] Audenaert A., Knockaert I., Collen D., Declerck P. (1994). Conversion of plasminogen activator inhibitor-1 from inhibitor to substrate by point mutations in the reactive-site loop. J. Biol. Chem..

[26] Van Meijer M., Smilde A., Tans G., Nesheim M.E., Pannekoek H., Horrevoets A.J. (1997). The Suicide Substrate Reaction between Plasminogen Activator Inhibitor 1 and Thrombin Is Regulated by the Cofactors Vitronectin and Heparin. Blood.

[27] Mottonen J., Strand A., Symerský J., Sweet R.M., Danley D.E., Geoghegan K.F., Gerard R.D., Goldsmith E.J. (1992). Structural basis of latency in plasminogen activator inhibitor-1. Nat. Cell Biol..

[28] Declerck P.J., De Mol M., Alessi M.C., Baudner S., Pâques E.P., Preissner K.T., Müller-Berghaus G., Collen D. (1988). Purification and characterization of a plasminogen activator inhibitor 1 binding protein from human plasma. Identification as a multimeric form of S protein (vitronectin). J. Biol. Chem..

[29] Lindahl T.L., Sigurdardottir O., Wiman B. (1989). Stability of Plasminogen Activator Inhibitor 1 (PAI-1). Thromb. Haemost..

[30] Jensen J.K., Thompson L.C., Bucci J.C., Nissen P., Gettins P.G.W., Peterson C.B., Andreasen P.A., Morth J.P. (2011). Crystal Structure of Plasminogen Activator Inhibitor-1 in an Active Conformation with Normal Thermodynamic Stability. J. Biol. Chem..

[31] Thompson L.C., Goswami S., Ginsberg D.S., Day D.E., Verhamme I.M., Peterson C.B. (2010). Metals affect the structure and activity of human plasminogen activator inhibitor-1. I. Modulation of stability and protease inhibition. Protein Sci..

[32] Keijer J., Linders M., Ehrlich H., Gebbink R.K., Pannekoek H. (1990). Stabilisation of plasminogen activator inhibitor type 1 (PAI-1) activity by arginine: Possible implications for the interaction of PAI-1 with vitronectin. Fibrinolysis.

[33] Mimuro J., Schleef R.R., Loskutoff D.J. (1987). Extracellular matrix of cultured bovine aortic endothelial cells contains functionally active type 1 plasminogen activator inhibitor. Blood.

[34] Smolarczyk K., Gils A., Boncela J., Declerck P.J., Cierniewski C.S. (2005). Function-Stabilizing Mechanism of Plasminogen Activator Inhibitor Type 1 Induced upon Binding to α1-Acid Glycoprotein. Biochemestry.

[35] Sillen M., Weeks S.D., Strelkov S.V., Declerck P.J. (2020). Structural Insights into the Mechanism of a Nanobody That Stabilizes PAI-1 and Modulates Its Activity. Int. J. Mol. Sci..

[36] De Taeye B., Gils A., Declerck P.J. (2004). The story of the serpin plasminogen activator inhibitor 1: Is there any need for another mutant?. Thromb. Haemost..

[37] Sillen M., Declerck P.J. (2020). Targeting PAI-1 in Cardiovascular Disease: Structural Insights Into PAI-1 Functionality and Inhibition. Front. Cardiovasc. Med..

[38] Bae H.-B., Zmijewski J.W., Deshane J.S., Zhi D., Thompson L.C., Peterson C.B., Chaplin D.D., Abraham E. (2012). Vitronectin Inhibits Neutrophil Apoptosis through Activation of Integrin-Associated Signaling Pathways. Am. J. Respir. Cell Mol. Biol..

[39] Wheaton A.K., Velikoff M., Agarwal M., Loo T.T., Horowitz J.C., Sisson T.H., Kim K.K. (2016). The vitronectin RGD motif regulates TGF-β-induced alveolar epithelial cell apoptosis. Am. J. Physiol. Cell. Mol. Physiol..

[40] Gettins P.G.W., Dolmer K. (2016). The High Affinity Binding Site on Plasminogen Activator Inhibitor-1 (PAI-1) for the Low Density Lipoprotein Receptor-related Protein (LRP1) Is Composed of Four Basic Residues. J. Biol. Chem..

[41] WHO (2018). Global Health Estimates 2016: Deaths by Cause, Age, Sex, by Country and by Region, 2000–2016.

[42] Erickson L.A., Fici G.J., Lund J.E., Boyle T.P., Polites H.G., Marotti K.R. (1990). Development of venous occlusions in mice transgenic for the plasminogen activator inhibitor-1 gene. Nat. Cell Biol..

[43] Eren M., Painter C.A., Atkinson J.B., Declerck P.J., Vaughan D.E. (2002). Age-Dependent Spontaneous Coronary Arterial Thrombosis in Transgenic Mice That Express a Stable Form of Human Plasminogen Activator Inhibitor-1. Circulation.

[44] Frangogiannis N.G. (2015). Pathophysiology of Myocardial Infarction. Compr. Physiol..

[45] Schneiderman J., Sawdey M.S., Keeton M.R., Bordin G.M., Bernstein E.F., Dilley R.B., Loskutoff D.J. (1992). Increased type 1 plasminogen activator inhibitor gene expression in atherosclerotic human arteries. Proc. Natl. Acad. Sci. USA.

[46] Lupu F., Bergonzelli G.E., Heim D.A., Cousin E., Genton C.Y., Bachmann F., Kruithof E.K. (1993). Localization and production of plasminogen activator inhibitor-1 in human healthy and atherosclerotic arteries. Arterioscler. Thromb. J. Vasc. Biol..

[47] Padró T., Steins M., Li C.-X., Mesters R.M., Hammel D., Scheld H.H., Kienast J. (1997). Comparative analysis of plasminogen activator inhibitor-1 expression in different types of atherosclerotic lesions in coronary arteries from human heart explants. Cardiovasc. Res..

[48] Rylander A.-C.J., Lindgren A., Deinum J., Bergström G.M.L., Böttcher G., Kalies I., Wåhlander K. (2017). Fibrinolysis inhibitors in plaque stability: A morphological association of PAI-1 and TAFI in advanced carotid plaque. J. Thromb. Haemost..

[49] Khoukaz H.B., Ji Y., Braet D.J., Vadali M., Abdelhamid A.A., Emal C.D., Lawrence D.A., Fay W.P. (2020). Drug Targeting of Plasminogen Activator Inhibitor-1 Inhibits Metabolic Dysfunction and Atherosclerosis in a Murine Model of Metabolic Syndrome. Arterioscler. Thromb. Vasc. Biol..

[50] Hamsten A., Wiman B., De Faire U., Blombäck M. (1985). Increased Plasma Levels of a Rapid Inhibitor of Tissue Plasminogen Activator in Young Survivors of Myocardial Infarction. N. Engl. J. Med..

[51] Tofler G., Massaro J., O’Donnell C., Wilson P., Vasan R., Sutherland P., Meigs J., Levy D., D’Agostino R. (2016). Plasminogen activator inhibitor and the risk of cardiovascular disease: The Framingham Heart Study. Thromb. Res..

[52] Jung R.G., Motazedian P., Ramirez F.D., Simard T., Di Santo P., Visintini S., Faraz M.A., Labinaz A., Jung Y., Hibbert B. (2018). Association between plasminogen activator inhibitor-1 and cardiovascular events: A systematic review and meta-analysis. Thromb. J..

[53] Song C., Burgess S., Eicher J.D., O’Donnell C.J., Johnson A.D., Huang J., Sabater-Lleal M., Asselbergs F.W., Tregouet D., Shin S. (2017). Causal Effect of Plasminogen Activator Inhibitor Type 1 on Coronary Heart Disease. J. Am. Heart Assoc..

[54] Meltzer M.E., Lisman T., De Groot P.G., Meijers J.C.M., Le Cessie S., Doggen C.J.M., Rosendaal F.R. (2010). Venous thrombosis risk associated with plasma hypofibrinolysis is explained by elevated plasma levels of TAFI and PAI-1. Blood.

[55] Chomiki N., Henry M., Alessi M.C., Anfosso F., Juhan-Vague I. (1994). Plasminogen Activator Inhibitor-1 Expression in Human Liver and Healthy or Atherosclerotic Vessel Walls. Thromb. Haemost..

[56] Juhan-Vague I., Alessi M.C., Vague P. (1991). Increased plasma plasminogen activator inhibitor 1 levels. A possible link between insulin resistance and atherothrombosis. Diabetology.

[57] Carratala A., Martinez-Hervas S., Rodriguez-Borja E., Benito E., Real J.T., Saez G.T., Carmena R., Ascaso J.F. (2017). PAI-1 levels are related to insulin resistance and carotid atherosclerosis in subjects with familial combined hyperlipidemia. J. Investig. Med..

[58] Ploplis V.A. (2011). Effects of altered plasminogen activator inhibitor-1 expression on cardiovascular disease. Curr. Drug Targets.

[59] Festa A., D’Agostino R., Tracy R.P., Haffner S.M. (2002). Elevated Levels of Acute-Phase Proteins and Plasminogen Activator Inhibitor-1 Predict the Development of Type 2 Diabetes: The Insulin Resistance Atherosclerosis Study. Diabetes.

[60] Yarmolinsky J., Barbieri N.B., Weinmann T., Ziegelmann P.K., Duncan B.B., Schmidt M.I. (2016). Plasminogen activator inhibitor-1 and type 2 diabetes: A systematic review and meta-analysis of observational studies. Sci. Rep..

[61] Alessi M.-C., Poggi M., Juhan-Vague I. (2007). Plasminogen activator inhibitor-1, adipose tissue and insulin resistance. Curr. Opin. Lipidol..

[62] Aguilar-Salinas C.A., Viveros-Ruiz T. (2019). Recent advances in managing/understanding the metabolic syndrome. F1000Research.

[63] Ninomiya T., Kubo M., Doi Y., Yonemoto K., Tanizaki Y., Rahman M., Arima H., Tsuryuya K., Iida M., Kiyohara Y. (2007). Impact of Metabolic Syndrome on the Development of Cardiovascular Disease in a General Japanese Population. Stroke.

[64] Iwasaki H., Okamoto R., Kato S., Konishi K., Mizutani H., Yamada N., Isaka N., Nakano T., Ito M. (2008). High glucose induces plasminogen activator inhibitor-1 expression through Rho/Rho-kinase-mediated NF-κB activation in bovine aortic endothelial cells. Atherosclerosis.

[65] Alessi M.C., Juhan-Vague I., Kooistra T., Declerck P.J., Collen D. (1988). Insulin Stimulates the Synthesis of Plasminogen Activator Inhibitor 1 by the Human Hepatocellular Cell Line Hep G2. Thromb. Haemost..

[66] Schneider D.J., Sobel B.E. (1996). Synergistic augmentation of expression of plasminogen activator inhibitor type-1 induced by insulin, very-low-density lipoproteins, and fatty acids. Coron. Artery Dis..

[67] Nordt T.K., Schneider D.J., Sobel B.E. (1994). Augmentation of the synthesis of plasminogen activator inhibitor type-1 by precursors of insulin. A potential risk factor for vascular disease. Circulation.

[68] Chen Y., Billadello J.J., Schneider D.J. (2000). Identification and localization of a fatty acid response region in the human plasminogen activator inhibitor-1 gene. Arterioscler. Thromb. Vasc. Biol..

[69] Fattal P., Schneider D., Sobel B., Billadello J. (1992). Post-transcriptional regulation of expression of plasminogen activator inhibitor type 1 mRNA by insulin and insulin-like growth factor 1. J. Biol. Chem..

[70] Sawdey M.S., Loskutoff D.J. (1991). Regulation of murine type 1 plasminogen activator inhibitor gene expression in vivo. Tissue specificity and induction by lipopolysaccharide, tumor necrosis factor-alpha, and transforming growth factor-beta. J. Clin. Investig..

[71] Alessi M.C., Peiretti F., Morange P., Henry M., Nalbone G., Juhan-Vague I. (1997). Production of Plasminogen Activator Inhibitor 1 by Human Adipose Tissue: Possible Link Between Visceral Fat Accumulation and Vascular Disease. Diabetes.

[72] Ellulu M.S., Patimah I., Khaza’Ai H., Rahmat A., Abed Y. (2017). Obesity and inflammation: The linking mechanism and the complications. Arch. Med. Sci..

[73] Pandey M., Loskutoff D.J., Samad F. (2005). Molecular mechanisms of tumor necrosis factor-α-mediated plasminogen activator inhibitor-1 expression in adipocytes. FASEB J..

[74] Rega G., Kaun C., Weiss T., Demyanets S., Zorn G., Kastl S., Steiner S., Seidinger D., Kopp C., Frey M. (2005). Inflammatory Cytokines Interleukin-6 and Oncostatin M Induce Plasminogen Activator Inhibitor-1 in Human Adipose Tissue. Circulation.

[75] Wang L., Chen L., Liu Z., Liu Y., Luo M., Chen N., Deng X., Luo Y., He J., Zhang L. (2018). PAI-1 Exacerbates White Adipose Tissue Dysfunction and Metabolic Dysregulation in High Fat Diet-Induced Obesity. Front. Pharmacol..

[76] Somodi S., Seres I., Lőrincz H., Harangi M., Fülöp P., Paragh G. (2018). Plasminogen Activator Inhibitor-1 Level Correlates with Lipoprotein Subfractions in Obese Nondiabetic Subjects. Int. J. Endocrinol..

[77] Levine J.A., Oleaga C., Eren M., Amaral A.P., Shang M., Lux E., Khan S.S., Shah S.J., Omura Y., Pamir N. (2021). Role of PAI-1 in hepatic steatosis and dyslipidemia. Sci. Rep..

[78] Morange P.E., Lijnen H.R., Alessi M.C., Kopp F., Collen D., Juhan-Vague I. (2000). Influence of PAI-1 on Adipose Tissue Growth and Metabolic Parameters in a Murine Model of Diet-Induced Obesity. Arterioscler. Thromb. Vasc. Biol..

[79] Schäfer K., Fujisawa K., Konstantinides S., Loskutoff D.J. (2001). Disruption of the plasminogen activator inhibitor-1 gene reduces the adiposity and improves the metabolic profile of genetically obese and diabetic ob/ob mice. FASEB J..

[80] Ma L.-J., Mao S.-L., Taylor K.L., Kanjanabuch T., Guan Y., Zhang Y., Brown N.J., Swift L.L., McGuinness O.P., Wasserman D.H. (2004). Prevention of Obesity and Insulin Resistance in Mice Lacking Plasminogen Activator Inhibitor 1. Diabetes.

[81] Henkel A.S., Khan S.S., Olivares S., Miyata T., Vaughan D.E. (2018). Inhibition of Plasminogen Activator Inhibitor 1 Attenuates Hepatic Steatosis but Does Not Prevent Progressive Nonalcoholic Steatohepatitis in Mice. Hepatol. Commun..

[82] Jensen L., Sloth B., Krog-Mikkelsen I., Flint A., Raben A., Tholstrup T., Brünner N., Astrup A. (2008). A low-glycemic-index diet reduces plasma plasminogen activator inhibitor-1 activity, but not tissue inhibitor of proteinases-1 or plasminogen activator inhibitor-1 protein, in overweight women. Am. J. Clin. Nutr..

[83] Lim J.H., Woo C.-H., Li J.-D. (2011). Critical role of type 1 plasminogen activator inhibitor (PAI-1) in early host defense against nontypeable Haemophilus influenzae (NTHi) infection. Biochem. Biophys. Res. Commun..

[84] Goolaerts A., LaFargue M., Song Y., Miyazawa B., Arjomandi M., Carlès M., Roux J., Howard M., Parks D.A., Iles K.E. (2011). PAI-1 is an essential component of the pulmonary host response during Pseudomonas aeruginosa pneumonia in mice. Thorax.

[85] Kager L.M., Van Der Windt G.J., Wieland C.W., Florquin S., van’t Veer C., Van Der Poll T. (2012). Plasminogen activator inhibitor type I may contribute to transient, non-specific changes in immunity in the subacute phase of murine tuberculosis. Microbes Infect..

[86] Matthay M.A., Ware L.B., Zimmerman G.A. (2012). The acute respiratory distress syndrome. J. Clin. Investig..

[87] Ozolina A., Sarkele M., Sabelnikovs O., Skesters A., Jaunalksne I., Serova J., Ievins T., Bjertnaes L.J., Vanags I. (2016). Activation of Coagulation and Fibrinolysis in Acute Respiratory Distress Syndrome: A Prospective Pilot Study. Front. Med..

[88] Kang S., Tanaka T., Inoue H., Ono C., Hashimoto S., Kioi Y., Matsumoto H., Matsuura H., Matsubara T., Shimizu K. (2020). IL-6 trans-signaling induces plasminogen activator inhibitor-1 from vascular endothelial cells in cytokine release syndrome. Proc. Natl. Acad. Sci. USA.

[89] Del Valle D.M., Kim-Schulze S., Huang H.-H., Beckmann N.D., Nirenberg S., Wang B., Lavin Y., Swartz T.H., Madduri D., Stock A. (2020). An inflammatory cytokine signature predicts COVID-19 severity and survival. Nat. Med..

[90] Tsantes A.E., Frantzeskaki F., Tsantes A.G., Rapti E., Rizos M., Kokoris S.I., Paramythiotou E., Katsadiotis G., Karali V., Flevari A. (2020). The haemostatic profile in critically ill COVID-19 patients receiving therapeutic anticoagulant therapy. Medicine.

[91] Kwaan H., Lindholm P. (2021). The Central Role of Fibrinolytic Response in COVID-19—A Hematologist’s Perspective. Int. J. Mol. Sci..

[92] Mahmood N., Mihalcioiu C., Rabbani S.A. (2018). Multifaceted Role of the Urokinase-Type Plasminogen Activator (uPA) and Its Receptor (uPAR): Diagnostic, Prognostic, and Therapeutic Applications. Front. Oncol..

[93] Devy L., Blacher S., Grignet-Debrus C., Bajou K., Masson V., Gerard R.D., Gils A., Carmeliet G., Carmeliet P., Declerck P.J. (2001). The pro- or antiangiogenic effect of plasminogen activator inhibitor 1 is dose dependent. FASEB J..

[94] Fang H., Placencio V.R., Declerck Y.A. (2012). Protumorigenic Activity of Plasminogen Activator Inhibitor-1 Through an Antiapoptotic Function. J. Natl. Cancer Inst..

[95] Balsara R.D., Ploplis V.A. (2008). Plasminogen activator inhibitor-1: The double-edged sword in apoptosis. Thromb. Haemost..

[96] Bajou K., Noel A., Gerard R.D., Masson V., Brunner N., Holsthansen C., Skobe M., Fusenig N.E., Carmeliet P., Collen D. (1998). Absence of host plasminogen activator inhibitor 1 prevents cancer invasion and vascularization. Nat. Med..

[97] Kubala M.H., Declerck Y.A. (2019). The plasminogen activator inhibitor-1 paradox in cancer: A mechanistic understanding. Cancer Metastasis Rev..

[98] Bajou K., Maillard C., Jost M., Lijnen R.H., Gils A., Declerck P., Carmeliet P., Foidart J.-M., Noël A. (2004). Host-derived plasminogen activator inhibitor-1 (PAI-1) concentration is critical for in vivo tumoral angiogenesis and growth. Oncogene.

[99] Isogai C., Laug W.E., Shimada H., Declerck P.J., Stins M.F., Durden D.L., Erdreich-Epstein A., Declerck Y.A. (2001). Plasminogen activator inhibitor-1 promotes angiogenesis by stimulating endothelial cell migration toward fibronectin. Cancer Res..

[100] Li H., Shinohara E., Cai Q., Chen H., Courtney R., Cao C., Wang Z., Teng M., Zheng W., Lu B. (2006). Plasminogen Activator Inhibitor-1 Promoter Polymorphism is Not Associated with the Aggressiveness of Disease in Prostate Cancer. Clin. Oncol..

[101] Błasiak J., Smolarz B. (2000). Plasminogen activator inhibitor-1 (PAI-1) gene 4G/5G promoter polymorphism is not associated with breast cancer. Acta Biochim. Pol..

[102] Sternlicht M.D., Dunning A.M., Moore D.H., Pharoah P.D., Ginzinger D.G., Chin K., Gray J.W., Waldman F.M., Ponder B.A., Werb Z. (2006). Prognostic Value of PAI1 in Invasive Breast Cancer: Evidence That Tumor-Specific Factors Are More Important Than Genetic Variation in Regulating PAI1 Expression. Cancer Epidemiol. Biomark. Prev..

[103] Jevrić M., Matić I.Z., Krivokuća A., Đorđić Crnogorac M., Besu I., Damjanović A., Branković-Magić M., Milovanović Z., Gavrilović D., Susnjar S. (2019). Association of uPA and PAI-1 tumor levels and 4G/5G variants of PAI-1 gene with disease outcome in luminal HER2-negative node-negative breast cancer patients treated with adjuvant endocrine therapy. BMC Cancer.

[104] Duffy M.J., McGowan P.M., Harbeck N., Thomssen C., Schmitt M. (2014). uPA and PAI-1 as biomarkers in breast cancer: Validated for clinical use in level-of-evidence-1 studies. Breast Cancer Res..

[105] Duffy M.J., O’Donovan N., McDermott E., Crown J. (2016). Validated biomarkers: The key to precision treatment in patients with breast cancer. Breast.

[106] Kates R.E., Gauger K., Willems A., Kiechle M., Magdolen V., Schmitt M., Harbeck N. (2004). Urokinase-type plasminogen activator (uPA) and its inhibitor PAI-1: Novel tumor-derived factors with a high prognostic and predictive impact in breast cancer. Thromb. Haemost..

[107] Nakatsuka E., Sawada K., Nakamura K., Yoshimura A., Kinose Y., Kodama M., Hashimoto K., Mabuchi S., Makino H., Morii E. (2017). Plasminogen activator inhibitor-1 is an independent prognostic factor of ovarian cancer and IMD-4482, a novel plasminogen activator inhibitor-1 inhibitor, inhibits ovarian cancer peritoneal dissemination. Oncotarget.

[108] Chan O.T., Furuya H., Pagano I., Shimizu Y., Hokutan K., Dyrskjøt L., Jensen J.B., Malmstrom P.-U., Segersten U., Janku F. (2017). Association of MMP-2, RB and PAI-1 with decreased recurrence-free survival and overall survival in bladder cancer patients. Oncotarget.

[109] Becker M., Szarvas T., Wittschier M., Dorp F.V., Tötsch M., Schmid K.W., Rübben H., Ergün S. (2010). Prognostic impact of plasminogen activator inhibitor type 1 expression in bladder cancer. Cancer.

[110] Sakakibara T., Hibi K., Koike M., Fujiwara M., Kodera Y., Ito K., Nakao A. (2005). Plasminogen activator inhibitor-1 as a potential marker for the malignancy of colorectal cancer. Br. J. Cancer.

[111] Zubac D.P., Wentzel-Larsen T., Seidal T., Bostad L. (2010). Type 1 plasminogen activator inhibitor (PAI-1) in clear cell renal cell carcinoma (CCRCC) and its impact on angiogenesis, progression and patient survival after radical nephrectomy. BMC Urol..

[112] Sotiropoulos G., Kotopouli M., Karampela I., Christodoulatos G.S., Antonakos G., Marinou I., Vogiatzakis E., Lekka A., Papavassiliou A., Dalamaga M. (2019). Circulating plasminogen activator inhibitor-1 activity: A biomarker for resectable non-small cell lung cancer?. J. BU ON Off. J. Balk. Union Oncol..

[113] Ghosh A.K., Vaughan D.E. (2011). PAI-1 in tissue fibrosis. J. Cell. Physiol..

[114] Lemaire R., Burwell T., Sun H., Delaney T., Bakken J., Cheng L., Rebelatto M.C., Czapiga M., De-Mendez I., Coyle A.J. (2015). Resolution of Skin Fibrosis by Neutralization of the Antifibrinolytic Function of Plasminogen Activator Inhibitor 1. Arthritis Rheumatol..

[115] Sun Y., Wang Y., Long J., Wang X. (2014). Association of the Plasminogen Activator Inhibitor-1 (PAI-1) Gene -675 4G/5G and -844 A/G Promoter Polymorphism with Risk of Keloid in a Chinese Han Population. Med. Sci. Monit. Int. Med. J. Exp. Clin. Res..

[116] Liu R.-M. (2008). Oxidative Stress, Plasminogen Activator Inhibitor 1, and Lung Fibrosis. Antioxid. Redox Signal..

[117] Courey A.J., Horowitz J.C., Kim K.K., Koh T.J., Novak M.L., Subbotina N., Warnock M., Xue B., Cunningham A.K., Lin Y. (2011). The vitronectin-binding function of PAI-1 exacerbates lung fibrosis in mice. Blood.

[118] Małgorzewicz S., Skrzypczak-Jankun E., Jankun J. (2013). Plasminogen activator inhibitor-1 in kidney pathology. Int. J. Mol. Med..

[119] Bergheim I., Guo L., Davis M.A., Duveau I., Arteel G.E. (2005). Critical Role of Plasminogen Activator Inhibitor-1 in Cholestatic Liver Injury and Fibrosis. J. Pharmacol. Exp. Ther..

[120] Chan J.C., Duszczyszyn D.A., Castellino F.J., Ploplis V.A. (2001). Accelerated Skin Wound Healing in Plasminogen Activator Inhibitor-1-Deficient Mice. Am. J. Pathol..

[121] Shioya S., Masuda T., Senoo T., Horimasu Y., Miyamoto S., Nakashima T., Iwamoto H., Fujitaka K., Hamada H., Hattori N. (2018). Plasminogen activator inhibitor-1 serves an important role in radiation-induced pulmonary fibrosis. Exp. Ther. Med..

[122] Huang W.-T., Vayalil P.K., Miyata T., Hagood J., Liu R.-M. (2012). Therapeutic Value of Small Molecule Inhibitor to Plasminogen Activator Inhibitor–1 for Lung Fibrosis. Am. J. Respir. Cell Mol. Biol..

[123] Senoo T., Hattori N., Tanimoto T., Furonaka M., Ishikawa N., Fujitaka K., Haruta Y., Murai H., Yokoyama A., Kohno N. (2010). Suppression of plasminogen activator inhibitor-1 by RNA interference attenuates pulmonary fibrosis. Thorax.

[124] Lassila M., Fukami K., Jandeleit-Dahm K., Semple T., Carmeliet P., Cooper M.E., Kitching A.R. (2007). Plasminogen activator inhibitor-1 production is pathogenetic in experimental murine diabetic renal disease. Diabetologia.

[125] Noguchi R., Kaji K., Namisaki T., Moriya K., Kawaratani H., Kitade M., Takaya H., Aihara Y., Douhara A., Asada K. (2020). Novel oral plasminogen activator inhibitor-1 inhibitor TM5275 attenuates hepatic fibrosis under metabolic syndrome via suppression of activated hepatic stellate cells in rats. Mol. Med. Rep..

[126] Xu Z., Castellino F.J., Ploplis V.A. (2010). Plasminogen activator inhibitor-1 (PAI-1) is cardioprotective in mice by maintaining microvascular integrity and cardiac architecture. Blood.

[127] Pedroja B.S., Kang L.E., Imas A.O., Carmeliet P., Bernstein A.M. (2009). Plasminogen Activator Inhibitor-1 Regulates Integrin αvβ3 Expression and Autocrine Transforming Growth Factor β Signaling. J. Biol. Chem..

[128] Soeda S., Koyanagi S., Kuramoto Y., Kimura M., Oda M., Kozako T., Hayashida S., Shimeno H. (2008). Anti-apoptotic roles of plasminogen activator inhibitor-1 as a neurotrophic factor in the central nervous system. Thromb. Haemost..

[129] Jeon H., Kim J.-H., Kim J.-H., Lee W.-H., Lee M.-S., Suk K. (2012). Plasminogen activator inhibitor type 1 regulates microglial motility and phagocytic activity. J. Neuroinflamm..

[130] Rodríguez-Lorenzo S., Francisco D.M.F., Vos R., Hof B.V.H., Rijnsburger M., Schroten H., Ishikawa H., Beaino W., Bruggmann R., Kooij G. (2020). Altered secretory and neuroprotective function of the choroid plexus in progressive multiple sclerosis. Acta Neuropathol. Commun..

[131] Gverić D., Herrera B., Petzold A., Lawrence D.A., Cuzner M.L. (2003). Impaired fibrinolysis in multiple sclerosis: A role for tissue plasminogen activator inhibitors. Brain.

[132] Oh J., Lee H.-J., Song J.-H., Park S.I., Kim H. (2014). Plasminogen activator inhibitor-1 as an early potential diagnostic marker for Alzheimer’s disease. Exp. Gerontol..

[133] Jacobsen J.S., Comery T.A., Martone R.L., Elokdah H., Crandall D.L., Oganesian A., Aschmies S., Kirksey Y., Gonzales C., Xu J. (2008). Enhanced clearance of A in brain by sustaining the plasmin proteolysis cascade. Proc. Natl. Acad. Sci. USA.

[134] Pan H., Zhao Y., Zhai Z., Zheng J., Zhou Y., Zhai Q., Cao X., Tian J., Zhao L. (2018). Role of plasminogen activator inhibitor-1 in the diagnosis and prognosis of patients with Parkinson’s disease. Exp. Ther. Med..

[135] Reuland C.J., Church F.C. (2020). Synergy between plasminogen activator inhibitor-1, α-synuclein, and neuroinflammation in Parkinson’s disease. Med. Hypotheses.

[136] Yates R.L., Esiri M.M., Palace J., Jacobs B., Perera R., DeLuca G.C. (2017). Fibrin(ogen) and neurodegeneration in the progressive multiple sclerosis cortex. Ann. Neurol..

[137] Angelucci F., Čechová K., Průša R., Hort J. (2018). Amyloid beta soluble forms and plasminogen activation system in Alzheimer’s disease: Consequences on extracellular maturation of brain-derived neurotrophic factor and therapeutic implications. CNS Neurosci. Ther..

[138] López-Otín C., Blasco M.A., Partridge L., Serrano M., Kroemer G. (2013). The Hallmarks of Aging. Cell.

[139] Tchkonia T., Zhu Y., Van Deursen J., Campisi J., Kirkland J.L. (2013). Cellular senescence and the senescent secretory phenotype: Therapeutic opportunities. J. Clin. Investig..

[140] Özcan S., Alessio N., Acar M.B., Mert E., Omerli F., Peluso G., Galderisi U. (2016). Unbiased analysis of senescence associated secretory phenotype (SASP) to identify common components following different genotoxic stresses. Aging.

[141] Khan S.S., Shah S.J., Klyachko E., Baldridge A.S., Eren M., Place A.T., Aviv A., Puterman E., Lloyd-Jones D.M., Heiman M. (2017). A null mutation in *SERPINE1* protects against biological aging in humans. Sci. Adv..

[142] Vaughan D.E., Rai R., Khan S.S., Eren M., Ghosh A.K. (2017). Plasminogen Activator Inhibitor-1 Is a Marker and a Mediator of Senescence. Arteroscler. Thromb. Vasc. Biol..

[143] Ersoy C., Kiyici S., Budak F., Oral B., Guclu M., Duran C., Selimoglu H., Erturk E., Tuncel E., Imamoglu S. (2008). The effect of metformin treatment on VEGF and PAI-1 levels in obese type 2 diabetic patients. Diabetes Res. Clin. Pract..

[144] Brown N.J., Kumar S., Painter C.A., Vaughan D.E. (2002). ACE Inhibition Versus Angiotensin Type 1 Receptor Antagonism. Hypertension.

[145] Baluta M.M., Vintila M.M. (2015). PAI-1 Inhibition—Another Therapeutic Option for Cardiovascular Protection. Maedica.

[146] Fortenberry Y.M. (2013). Plasminogen activator inhibitor-1 inhibitors: A patent review (2006–present). Expert Opin. Ther. Pat..

[147] Rouch A., Vanucci-Bacqué C., Bedos-Belval F., Baltas M. (2015). Small molecules inhibitors of plasminogen activator inhibitor-1—An overview. Eur. J. Med. Chem..

[148] D’Amico S., Martial J.A., Struman I. (2012). A peptide mimicking the C-terminal part of the reactive center loop induces the transition to the latent form of plasminogen activator inhibitor type-1. FEBS Lett..

[149] Mathiasen L., Dupont D.M., Christensen A., Blouse G.E., Jensen J.K., Gils A., Declerck P.J., Wind T., Andreasen P.A. (2008). A Peptide Accelerating the Conversion of Plasminogen Activator Inhibitor-1 to an Inactive Latent State. Mol. Pharmacol..

[150] Jensen J.K., Malmendal A., Schiøtt B., Skeldal S., Pedersen K.E., Celik L., Nielsen N.C., Andreasen P.A., Wind T. (2006). Inhibition of plasminogen activator inhibitor-1 binding to endocytosis receptors of the low-density-lipoprotein receptor family by a peptide isolated from a phage display library. Biochem. J..

[151] Fjellström O., Deinum J., Sjögren T., Johansson C., Geschwindner S., Nerme V., Legnehed A., McPheat J., Olsson K., Bodin C. (2013). Characterization of a Small Molecule Inhibitor of Plasminogen Activator Inhibitor Type 1 That Accelerates the Transition into the Latent Conformation. J. Biol. Chem..

[152] Lin Z., Jensen J.K., Hong Z., Shi X., Hu L., Andreasen P.A., Huang M. (2013). Structural Insight into Inactivation of Plasminogen Activator Inhibitor-1 by a Small-Molecule Antagonist. Chem. Biol..

[153] Egelund R., Einholm A.P., Pedersen K.E., Nielsen R.W., Christensen A., Deinum J., Andreasen P.A. (2001). A Regulatory Hydrophobic Area in the Flexible Joint Region of Plasminogen Activator Inhibitor-1, Defined with Fluorescent Activity-neutralizing Ligands. J. Biol. Chem..

[154] Sillen M., Miyata T., Vaughan D., Strelkov S., Declerck P. (2021). Structural Insight into the Two-Step Mechanism of PAI-1 Inhibition by Small Molecule TM5484. Int. J. Mol. Sci..

[155] Li S.-H., Reinke A.A., Sanders K.L., Emal C.D., Whisstock J.C., Stuckey J.A., Lawrence D.A. (2013). Mechanistic characterization and crystal structure of a small molecule inactivator bound to plasminogen activator inhibitor-1. Proc. Natl. Acad. Sci. USA.

